# Culture Medium Composition and Light Intensity Shape Alternative Morphogenic Pathways from the Mature Embryo Shoot Apex of *Eragrostis curvula*

**DOI:** 10.3390/plants15182793

**Published:** 2026-09-11

**Authors:** Eduardo Daniel Souza Canada, Ingrid Garbus, Juan Pablo Selva, Hugo Raúl Permingeat, Viviana Echenique

**Affiliations:** 1 Plataforma AGROBIOTEC-FCA, Facultad de Ciencias Agrarias, Universidad Nacional de Rosario (UNR), Campo Experimental Villarino, Zavalla S2125ZAA, Santa Fe, Argentina; eduardo.souzacanada@unr.edu.ar; 2 Centro de Recursos Naturales Renovables de la Zona Semiárida/CERZOS, CCT CONICET Bahía Blanca, Camino La Carrindanga Km 7, Bahía Blanca 8000, Buenos Aires, Argentina; jpselva@criba.edu.ar; 3 Departamento de Ciencias de la Salud, Universidad Nacional del Sur (UNS), Florida 1450, Bahía Blanca 8000, Buenos Aires, Argentina; 4 Departamento de Agronomía, Universidad Nacional del Sur (UNS), San Andrés 800, Bahía Blanca 8000, Buenos Aires, Argentina; 5 IICAR-CONICET, Campo Experimental Villarino, Zavalla S2125ZAA, Santa Fe, Argentina

**Keywords:** weeping lovegrass, somatic embryo-like structures (SELSs), shoot apical meristem, light intensity, shoot multiplication, mature embryos, morphogenic responses

## Abstract

*Eragrostis curvula* (weeping lovegrass) is a perennial C_4_ forage grass and a model for apomixis whose genetic transformation and genome editing remain constrained by the lack of efficient regeneration systems. Here we assessed the effect of culture medium composition and light intensity during the induction phase on morphogenic responses from explants of mature embryos of cv. Tanganyika INTA. Eleven media were tested: nine designed to induce somatic embryo-like structures (SELSs; Group 1) and two to promote multiple shoot proliferation (Group 2). Four light regimes—low, medium, high, and darkness—were applied during an 8-week induction period. Responsive and non-responsive explants were recorded at the Petri dish level and analyzed separately within each functional group using binomial generalized linear models (GLMs) in R 4.5.0. Morphogenic responses originated from the apical region of the embryonic axis, encompassing the shoot apical meristem. Medium composition influenced the predominant morphogenic response, whereas light intensity affected the efficiency of the response (significant medium × light interaction). Medium I (WPBS + 2,4-D + low BAP) under medium light resulted in the highest morphogenic response within Group 1, corresponding to an estimated 60.6% of explants exhibiting SELSs and/or associated shoot-forming meristematic tissue, although this estimate was not statistically distinguishable from those obtained for the same medium under low and high light intensity. Medium J (WPBS + high BAP:2,4-D ratio) under medium light resulted in a 74.0% multiple shoot proliferation response. Darkness reduced morphogenic responses in both groups. These protocols provide a reproducible regeneration platform for future genetic transformation and genome-editing studies in *E. curvula*.

## 1. Introduction

*Eragrostis curvula* (Schrad.) Nees, commonly known as weeping lovegrass, is a perennial warm-season C_4_ grass native to southern Africa and naturalized across arid and semi-arid regions of South America, North America, and Australia. In Argentina, it covers millions of hectares and constitutes a key forage resource for extensive livestock systems in marginal environments owing to its drought tolerance, adaptation to poor soils, high biomass production, and erosion-control capacity [1]. Beyond its agronomic importance, *E. curvula* is also recognized as a model species for studying apomixis, since the genus includes both sexual diploids and apomictic polyploids [2].

Despite its importance, the application of modern biotechnological approaches to *E. curvula* has been limited by its recalcitrance to in vitro tissue culture. A transformation protocol was reported by Ncanana et al. [3] using biolistic bombardment of callus derived from leaf and seed explants of cv. Ermelo, achieving a stable transformation frequency of 13%. However, this protocol relied on organogenic regeneration and was developed using a single genotype. To date, no efficient, genotype-independent transformation protocol has been established for *E. curvula*, and the available protocol remains unsuitable for high-throughput applications [3]. The development of an efficient and reproducible regeneration system is therefore an essential prerequisite for improving transformation strategies, including biolistic delivery, *Agrobacterium*-mediated transformation, and CRISPR/Cas9-based genome editing [4,5].

The shoot apical meristem (SAM) is the central organizer of shoot architecture in grasses, maintaining a population of pluripotent stem cells [6]. In recent decades, the SAM has emerged as a sustainable explant for genetic transformation of cereal crops, offering a less genotype-dependent alternative to embryogenic callus derived from immature embryos [7]. Systems enabling multiple shoot proliferation from SAMs have been established in oat [8], wheat [9], and other cereals, demonstrating that SAMs from diverse species can be induced to form multiple shoots at high frequency. A further complication in *E. curvula* is that the apical region of the embryonic axis becomes covered by non-embryogenic tissue within the first few days of culture, thereby severely restricting the temporal window available for effective genetic manipulation. Similar limitations have been described in other recalcitrant cereals, in which SAM-based regeneration systems have provided an effective alternative to conventional embryo-derived callus approaches [7,9,10]. However, whether the same shoot apical region can be directed toward alternative morphogenic pathways depending on the inductive environment has not previously been investigated in *E. curvula*.

Mature embryos offer a practical alternative to immature embryos, as they are readily available year-round and have enabled rapid, genotype-independent shoot regeneration in other recalcitrant species [11]. This is particularly relevant in warm-season grasses, where the frequency of embryogenic callus formation from immature material is often low due to strong genetic heterogeneity, making mature seed-derived explants a more consistent starting material for these species [12]. The use of mature embryos has been successfully scaled up for commercial genetic transformation in maize [13], and direct transformation has been demonstrated in wheat [5]. Within the genus *Eragrostis*, Gugsa and Kumlehn [14] developed a highly effective protocol for somatic embryogenesis from immature zygotic embryos of tef (*Eragrostis tef*), yielding more than 2800 shoots per explant. More recently, Dalton [10] reformulated MS medium (WPBS medium) to improve embryogenesis in sixteen grass and cereal species.

The induction of somatic embryogenesis involves a complex interplay between stress signals and hormonal regulation [15]. The interaction of auxins (particularly 2,4-D) and cytokinins (such as BAP) is well-established as a key determinant of morphogenic pathways in vitro [9,10]. Light is also a critical environmental signal that influences plant morphogenesis in vitro [16,17,18]. Light intensity, spectral quality, and photoperiod represent distinct components of the light environment that can influence plant regeneration [18]. In the present study, light intensity was selected as the primary environmental variable to specifically evaluate the effect of irradiance during the induction phase, while the same fluorescent light source and a 16 h light/8 h dark photoperiod were maintained across illuminated treatments. This design allowed us to assess responses to different irradiance levels without simultaneously varying the light source or photoperiod. Nevertheless, information regarding the influence of light intensity during the induction phase remains scarce in perennial grasses, particularly when mature embryos are used as explants.

In this study, we evaluated the regeneration potential of *E. curvula* cv. Tanganyika INTA through the formation of somatic embryo-like structures and multiple shoot proliferation using mature embryos as the sole source of explants. We assessed eleven induction-medium formulations grouped into two functional categories under four light intensities and analyzed the resulting responses using generalized linear models. Our working hypothesis was that culture medium composition and light intensity interact during the induction phase to determine the morphogenic response and the subsequent developmental pathway. This work, therefore, aimed to determine whether culture medium composition and light intensity direct alternative morphogenic pathways from the shoot apical region of mature embryos, thereby providing a regeneration framework for future genetic transformation of *E. curvula*.

## 2. Results

### 2.1. Morphological Changes During Culture Establishment

**Figure 1:**
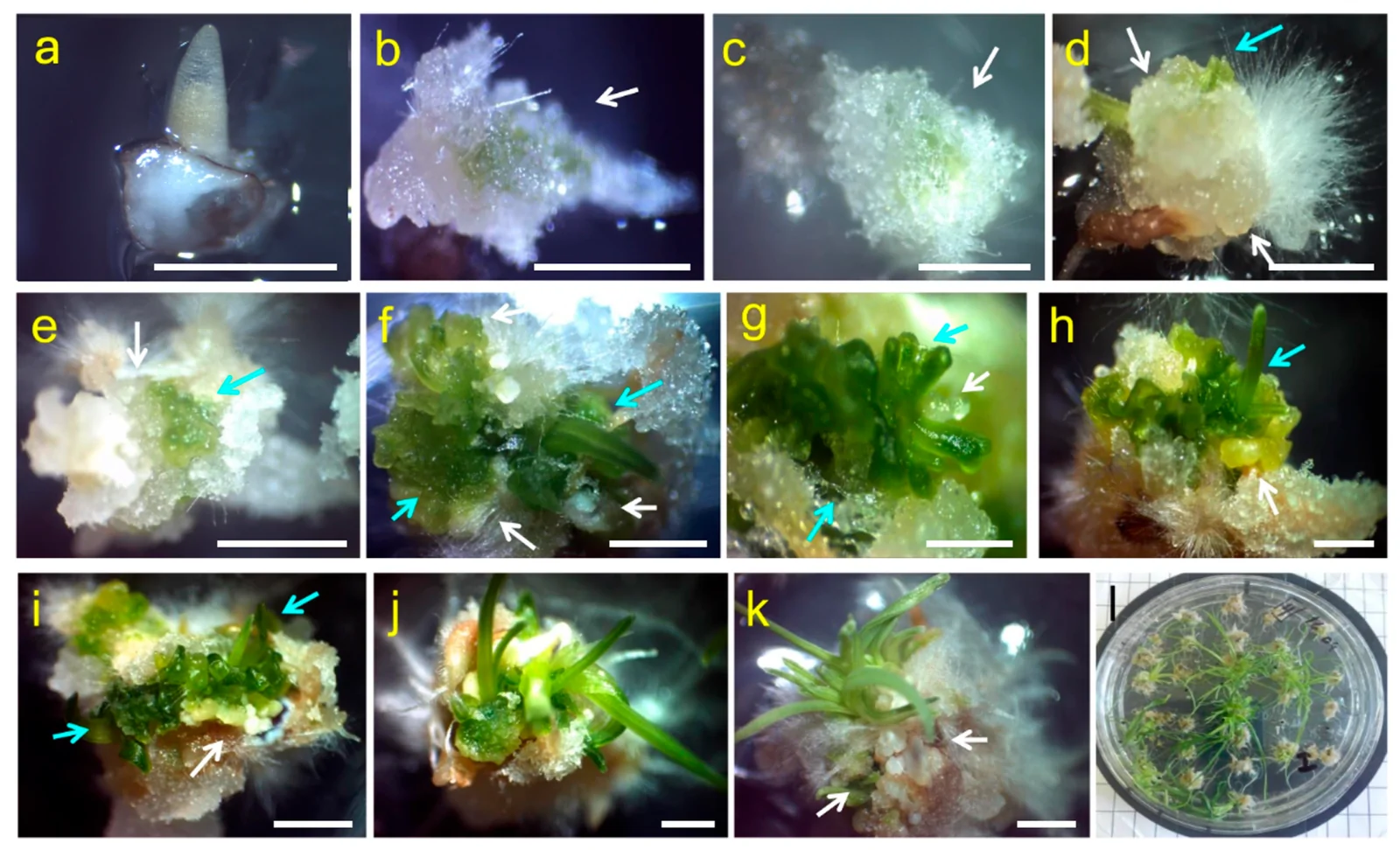
Sequential morphological changes of mature embryo explants of *Eragrostis curvula* cv. Tanganyika INTA cultured on Medium A under a light intensity of 55.2 µmol m^−2^·s^−1^. (**a**) Day 2: Initial elongation of the coleoptile and first leaf primordium, whereas the radicle region remained only weakly developed. (**b**,**c**) Days 8–14: Continued elongation of the coleoptile and first leaf primordium, with progressive proliferation of non-morphogenic tissue covering the apical region of the embryonic axis (white arrowheads). (**d**,**e**) Days 18–19: Appearance of compact, white to translucent globular structures, hereafter referred to as somatic embryo-like structures (SELSs; based on external morphology only) (white arrowheads), together with the first organized green meristematic regions (cyan arrows). (**f**) Day 25: Persistence of SELSs (white arrowheads) and progressive development of green shoot primordia (cyan arrows). (**g**,**h**) Days 30–32: Coexistence of arrested SELSs (white arrowheads) and actively developing green shoot clusters (cyan arrows). (**i**,**j**) Day 34: Continued development of organized multiple shoots. (**k**) Day 42: Well-developed regenerated plantlets with expanded leaves and root systems; residual SELSs (white arrowheads) remain associated with the base of the regenerating shoots. (**l**) Day 46: Overview of regenerated plantlets cultured in the same Petri dish. White scale bars = 2 mm.

Sequential observations of mature embryo explants of *Eragrostis curvula* cv. Tanganyika INTA cultured on Medium A under a light intensity of 55.2 µmol m^−2^·s^−1^ revealed reproducible morphogenic changes leading to complete plantlet regeneration within six weeks of culture (Figure 1).

During the first two days of culture on Medium A, under medium light intensity (55.2 µmol m^−2^·s^−1^), the earliest morphogenic response consisted of slight elongation of the coleoptile and first leaf primordium, while radicle growth remained limited (Figure 1a).

Between 8 and 14 days after culture initiation, elongation of the coleoptile continued, and proliferating non-morphogenic tissue progressively covered the apical region of the embryonic axis, while the scutellum showed no visible morphological changes (Figure 1b,c).

From approximately day 15 onwards, localized morphogenic activity became visible beneath the proliferating tissue. Two morphologically distinct types of structures subsequently emerged from the same anatomical region of the embryonic axis. The first consisted of compact, white to translucent globular bodies lacking visible chlorophyll pigmentation (Figure 1d,e). Based exclusively on their external morphology, these globular bodies are hereafter referred to as SELSs. The second response consisted of small green meristematic regions that appeared adjacent to these globular structures and progressively increased in size (Figure 1d,e).

During the fourth week of culture (22–28 days), both types of structures became more conspicuous. While the globular bodies increased slightly in diameter, they retained their compact external morphology without developing visible features indicative of the bipolar organization characteristic of mature somatic embryos. Because histological analysis was not performed, neither bipolar organization nor the presence or absence of vascular connection to the maternal tissue could be determined. In contrast, the green meristematic regions became progressively organized into shoot primordia showing increasing chlorophyll accumulation (Figure 1f). Between 29 and 42 days of culture, these green structures differentiated into dense clusters of multiple shoots and subsequently developed into complete plantlets with expanded leaves and typical green pigmentation (Figure 1g–j). By 42–46 days of culture, regenerated plantlets exhibited well-developed shoots and root systems, indicating complete plant regeneration (Figure 1k,l).

Importantly, the sequential photographic documentation consistently showed that both the globular structures and the organized green tissues arose from the apical region of the embryonic axis. No morphogenic response was observed on the scutellum surface under the conditions evaluated. Although the cellular origin of the induced structures could not be determined, the high reproducibility of this developmental sequence indicates that the responsive region is associated with the embryonic shoot apex. Additional representative photographs documenting the temporal progression of external morphogenic changes under this culture condition are provided in Figure S1.

### 2.2. Effect of Culture Medium and Light Intensity on Morphogenic Induction in Group 1 Media

The effects of culture medium composition and light intensity on morphogenic induction, defined as the formation of SELSs and/or shoot-forming meristematic tissue, were evaluated.

**Figure 2:**
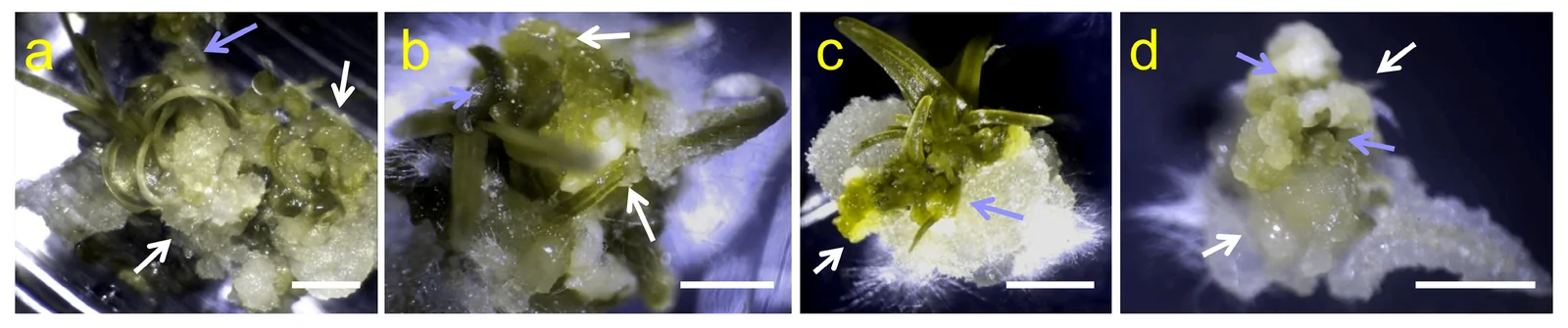
Representative examples of SELS-associated morphogenic responses obtained on Group 1 culture media under different light conditions. White arrowheads indicate compact white to translucent globular structures referred to as SELSs, whereas cyan arrows indicate developing shoot meristems. (**a**) Explants cultured on Medium I under medium light intensity (55.2 µmol m^−2^·s^−1^) after 52 days, illustrating abundant SELSs associated with actively developing shoot meristems under one of the treatments yielding the highest morphogenic response in Group 1. (**b**) Explants cultured on Medium A under medium light intensity (55.2 µmol m^−2^·s^−1^) after 26 days, showing SELS formation. (**c**) Explants cultured on Medium C under high light intensity after 30 days, showing abundant SELS formation. (**d**) Explants cultured on Medium F under low light intensity after 23 days, showing SELS formation and associated morphogenic structures in a medium containing 6% maltose. White scale bars = 2 mm.

Photographic observations showed that responsive explants from Group 1 media displayed broadly similar external morphogenic features despite quantitative differences in induction probability (Figure 2). Initial responses became evident during the third week of culture, when compact white or translucent globular structures appeared close to the apical region of the embryonic axis. These structures remained externally smooth and compact throughout the culture period and generally failed to progress toward visible embryo maturation.

In several of the media evaluated, these globular structures were frequently accompanied by adjacent green, shoot meristem-like structures (Figure 2a–d). While the embryo-like bodies remained morphologically unchanged throughout the induction period, the green meristematic structures continued their development toward shoot formation, suggesting that the same responsive region of the embryonic axis could give rise to two distinct morphogenic responses. From a plant-regeneration perspective, the development of organized shoot meristems represents the most relevant outcome, as these structures subsequently gave rise to complete regenerated plantlets. Additional representative images illustrating the morphological diversity observed across Group 1 culture media and light conditions are provided in Figure S2.

Generalized linear model (GLM) analysis revealed highly significant effects of culture medium (LR χ^2^ = 97.02; df = 8; *p* < 0.0001), light intensity (LR χ^2^ = 12.71; df = 3; *p* = 0.0053), and the interaction between the two factors (LR χ^2^ = 116.11; df = 24; *p* < 0.0001). These results indicate significant differences in morphogenic induction among culture media and across light intensities. Complete GLM outputs are provided in Supplementary Table S2.

### 2.3. Effect of Culture Medium and Light Intensity on Multiple-Shoot Proliferation (Group 2)

The effects of culture medium composition and light intensity on multiple-shoot proliferation were evaluated using Media J and K.

**Figure 3:**
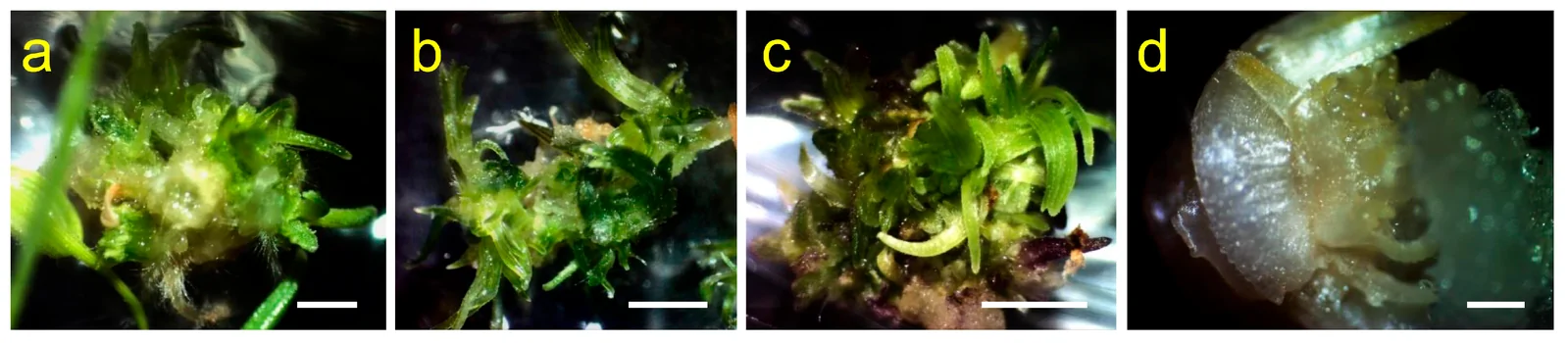
Representative examples of multiple-shoot proliferation observed on Group 2 induction media. (**a**) Explants cultured on Medium J under medium light intensity (55.2 µmol m^−2^·s^−1^) after 27 days, showing the highest shoot proliferation observed in this study. (**b**) Explants cultured on Medium J under low light intensity (29.4 µmol m^−2^·s^−1^) after 27 days, showing shoot cluster formation. (**c**) Explants cultured on Medium K under high light intensity (107.4 µmol m^−2^·s^−1^) after 48 days, showing dense shoot cluster formation. (**d**) Explants cultured on Medium J under darkness after 27 days, showing reduced shoot proliferation, consistent with the lower response observed under darkness. White scale bars = 2 mm.

Morphological observations revealed that explants cultured on Group 2 media exhibited a morphogenic response distinct from that observed in Group 1 (Figure 3). Rather than producing the globular SELSs characteristic of Group 1, the responsive region of the embryonic axis rapidly developed localized green meristematic tissue in Group 2 media that progressively differentiated into clusters of adventitious shoots. Well-defined, compact white to translucent SELSs were not a characteristic feature of Group 2 cultures.

Shoot primordia developed within a confined region corresponding to the apical portion of the embryonic axis and subsequently developed into dense shoot clusters that regenerated complete plantlets after transfer to the regeneration medium. In responsive explants, no conspicuous intervening callus phase was observed. Additional representative images illustrating multiple-shoot proliferation across Group 2 culture media and light conditions are provided in Figure S3.

Generalized linear model analysis revealed that light intensity (LR χ^2^ = 62.90; df = 3; *p* < 0.0001) and the culture medium × light intensity interaction (LR χ^2^ = 53.10; df = 3; *p* < 0.0001) had highly significant effects on multiple-shoot proliferation, whereas the main effect of culture medium was not significant (LR χ^2^ = 1.29; df = 1; *p* = 0.256). These results indicate significant effects of light intensity and its interaction with culture medium on multiple-shoot proliferation, whereas the main effect of culture medium was not significant. Complete GLM outputs are provided in Supplementary Table S3.

### 2.4. Comparative Analysis of Morphogenic Responses Across Culture Media and Light Environments

**Figure 4:**
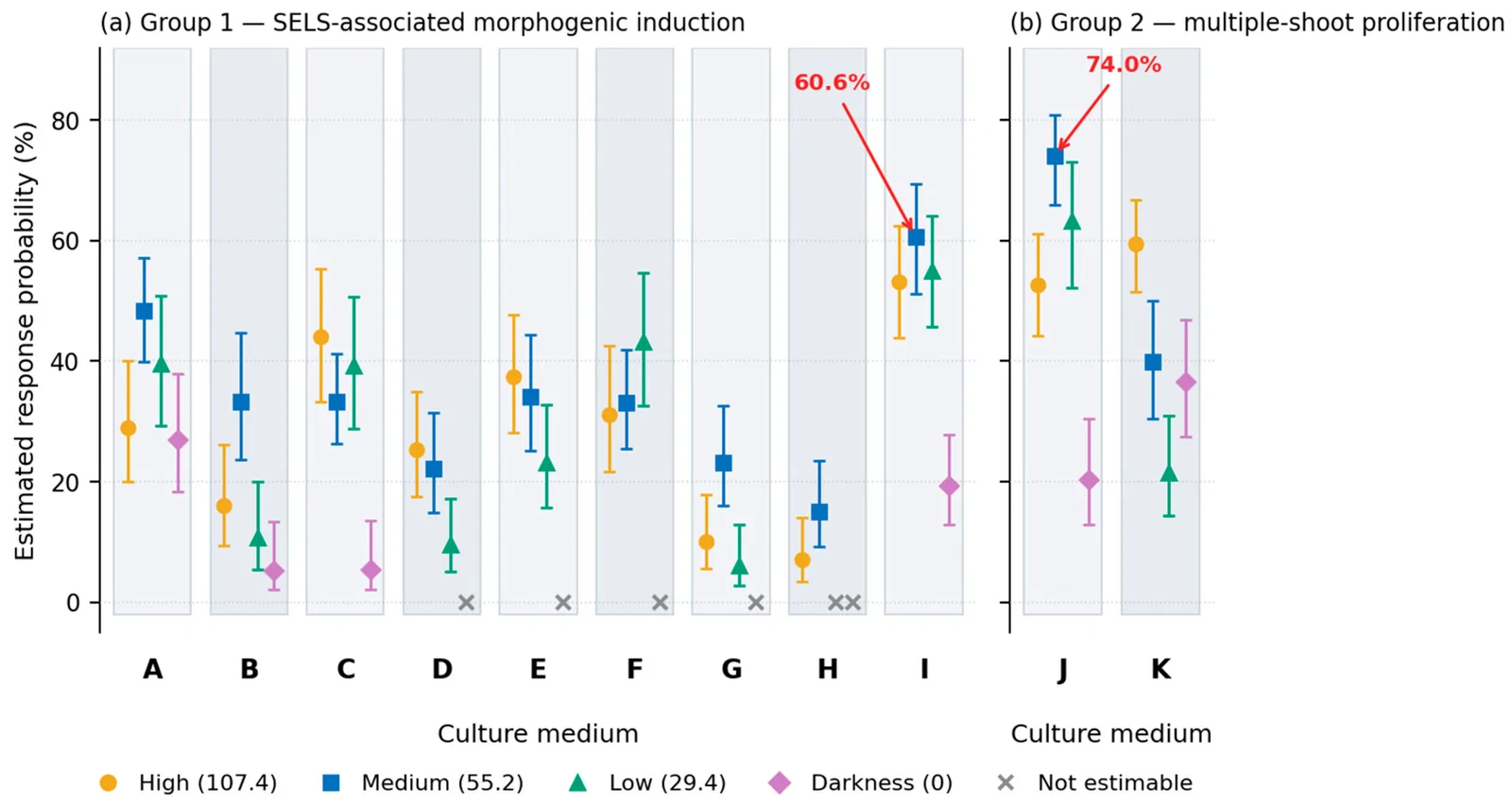
Estimated marginal means of predicted response probabilities obtained from separately fitted binomial generalized linear models, shown for each culture medium under the four light intensities evaluated. (**a**) Group 1 (Media A–I), in which responsiveness was defined as the formation of SELSs and/or associated shoot-forming meristematic tissue. (**b**) Group 2 (Media J and K), in which responsiveness was defined as multiple-shoot proliferation. Each shaded block groups the four light treatments applied to a single culture medium, indicated by the letter below the block. Symbols represent estimated marginal means on the response scale and whiskers indicate 95% confidence intervals. Crosses indicate culture medium × light combinations in which no responsive explants were recorded and for which complete separation resulted in boundary parameter estimates; model-based confidence intervals and pairwise comparisons were therefore not interpreted for these combinations. Red annotations indicate the highest estimated probability within each functional group. Light treatments were high light (107.4 µmol m^−2^·s^−1^), medium light (55.2 µmol m^−2^·s^−1^), low light (29.4 µmol m^−2^·s^−1^), and darkness. Complete GLM outputs, estimated marginal means with standard errors and 95% confidence intervals, and compact letter displays are provided in Supplementary Tables S2–S7.

The generalized linear models showed that culture medium composition and light intensity significantly influenced morphogenic responses in mature embryo explants. However, the two response types exhibited distinct response patterns. The unified presentation of estimated marginal means highlights these contrasting response profiles between the two functional groups despite their common anatomical origin (Figure 4).

For Group 1 media (A–I), morphogenic induction occurred over a broad range of formulations, although with markedly different efficiencies. Medium I showed the highest predicted induction probabilities among the illuminated treatments, reaching 60.6% under medium light intensity, followed by 55.1% under low light and 53.2% under high light intensity. These three illuminated treatments did not differ significantly from one another. Medium A under medium light (48.4%), Medium C under high light (44.0%) and Medium F under low light (43.2%) also exhibited relatively high predicted induction probabilities.

In contrast, Media G and H exhibited consistently low responses under most light conditions. Under darkness, morphogenic induction was markedly reduced or completely absent in several media. No responsive explants were observed in Media D, E, F, G and H under dark conditions. Overall, among the four light environments evaluated, medium light intensity generally produced the highest induction response in six of the nine Group 1 media tested, whereas darkness consistently reduced or abolished morphogenic induction.

Group 2 media (J and K) promoted multiple-shoot proliferation rather than abundant SELS formation (Figure 4). Medium J showed the highest predicted proliferation probability under medium light intensity (74.0%), followed by low light (63.3%), high light (52.7%) and darkness (20.2%). Medium K, in contrast, reached its highest predicted proliferation probability under high light intensity (59.4%), whereas substantially lower responses were obtained under medium light (39.8%), darkness (36.6%), and low light intensity (21.5%).

Overall, medium light intensity (55.2 µmol m^−2^·s^−1^) was associated with the highest morphogenic responses in both functional groups, although the magnitude of this effect depended on the culture medium.

Although their cellular origin remains to be determined histologically, these observations suggest that both morphogenic responses originate from the same morphogenically responsive domain of the embryonic axis but follow distinct developmental trajectories depending on the culture conditions. Complete estimated marginal means and compact letter displays for both groups are provided in Supplementary Tables S4–S7.

### 2.5. Summary of Morphogenic Responses: A Conceptual Model

**Figure 5:**
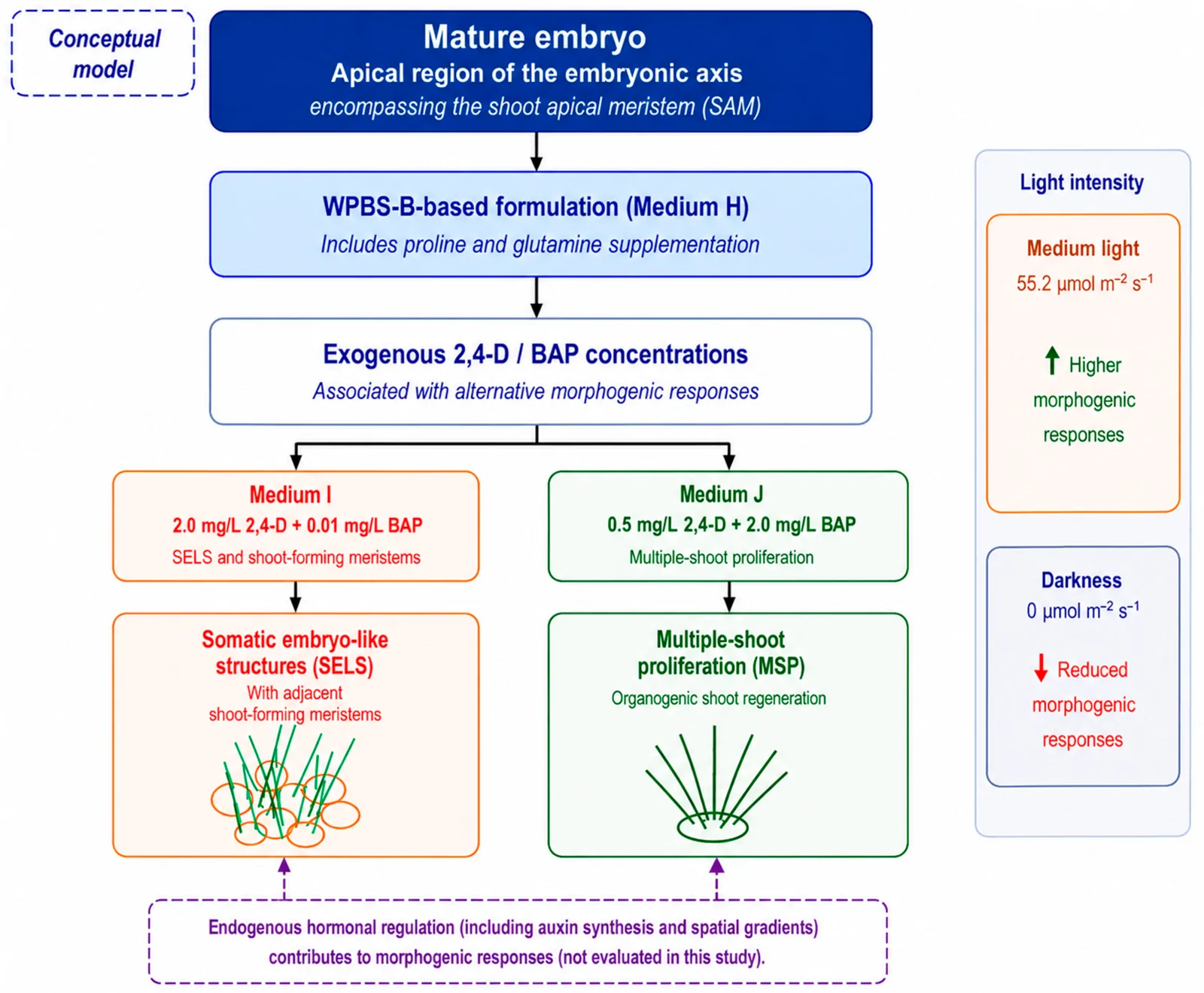
Conceptual model illustrating the morphogenic responses observed in mature embryos of *Eragrostis curvula* cv. Tanganyika INTA. The apical region of the embryonic axis, encompassing the shoot apical meristem (SAM), is represented as a morphogenically responsive domain. Different combinations of exogenous 2,4-D and BAP concentrations and light intensities were associated with distinct morphogenic responses, including somatic embryo-like structures (SELSs) and multiple-shoot proliferation. Higher response probabilities were observed under medium light (55.2 µmol m^−2^·s^−1^), whereas responses were reduced under darkness. The proposed conceptual model summarizes the associations observed among culture-medium formulation, exogenous 2,4-D and BAP concentrations, light intensity, and morphogenic responses in the present study. Endogenous hormonal regulation, including auxin synthesis and spatial gradients, is included as a conceptual component of the proposed morphogenic framework but was not evaluated experimentally in the present study [22]. In the SELS schematic, orange outlines represent somatic embryo-like structures (SELSs), whereas green lines represent adjacent shoot-forming meristems.

The combined morphological and statistical results support a conceptual model in which the apical region of the embryonic axis is considered a morphogenically responsive domain (Figure 5). Culture medium composition and light intensity were associated with two contrasting morphogenic responses: SELS-associated morphogenic induction in Group 1 and multiple-shoot proliferation in Group 2.

Comparable alternative morphogenic responses have been reported in other plant species. In cumin, embryo explants produced different morphogenic responses depending on the concentrations and combinations of exogenous plant growth regulators [19]. Similarly, mature caryopsis-derived cultures of *Pogonatherum paniceum* exhibited somatic embryogenesis, shoot organogenesis, or both depending on 2,4-D concentration [20], whereas the simultaneous occurrence of these responses was reported only rarely in sorghum explants [21].

The proposed conceptual model summarizes the associations observed among culture-medium formulation, exogenous 2,4-D and BAP concentrations, light intensity, and morphogenic responses.

## 3. Discussion

The present results indicate that the apical region of mature embryos of *Eragrostis curvula*, encompassing the shoot apical meristem, constitutes a highly responsive explant region whose morphogenic response is strongly associated with the interaction between culture medium composition and light intensity during induction. The central finding is that two distinct morphogenic responses, namely SELS-associated morphogenic induction and multiple-shoot proliferation, were observed under different combinations of culture medium and light intensity.

### 3.1. The Embryonic Shoot Apex of Mature Embryos Constitutes the Main Responsive Tissue

A central finding of this study is the identification of the apical region of the embryonic axis as the main responsive region. Sequential photographic documentation consistently showed that both SELSs and organized green shoot structures developed in association with the apical region of the embryonic axis, while no morphogenic response was observed on the scutellum under the conditions evaluated. This observation is consistent with findings in other cereals, where SAM-based regeneration systems have proven more effective than scutellum-derived callus approaches [7,9,10].

In *E. curvula,* the responsiveness of the embryo apex in mature embryos is particularly notable because the scutellum—the most used explant in grass tissue culture—failed to produce any morphogenic response. This contrasts with earlier studies in *Eragrostis tef*, where somatic embryogenesis was successfully induced from immature zygotic embryos via scutellum-derived callus [14]. The differences in responsiveness may reflect differences in embryo maturity and the developmental stage of the tissue at the time of excision.

Our results extend to *E. curvula* the role of the SAM as the central organizer of shoot architecture in grasses [6], showing that the embryonic shoot apex of mature embryos retains regenerative capacity and is associated with distinct morphogenic responses under the culture conditions evaluated. The persistence of globular SELSs and the continued development of green meristematic structures into shoots suggest that the embryonic shoot apex can support two distinct morphogenic responses that followed different developmental trajectories under the conditions evaluated [15].

### 3.2. The WPBS Formulation Supported the Highest Morphogenic Responses Observed in This Study

A key observation of this study is that the WPBS formulation provided the nutritional basis associated with the highest morphogenic responses. The WPBS-based Medium H (2,4-D, no BAP) induced only modest responses (15.0% under medium light), suggesting that the WPBS macronutrient formulation, with its mineral and amino acid composition [10], provides a nutritional context compatible with morphogenic competence without, by itself, favoring a specific developmental response. Within this context, the exogenous 2,4-D and BAP concentrations were associated with SELS-associated morphogenic induction in Medium I or multiple-shoot proliferation in Medium J. The WPBS formulation, originally developed by Dalton [10], modifies MS medium by increasing magnesium, phosphate, sulfate, copper, proline, and glutamine concentrations while reducing macronutrients to three-quarter strength. The inclusion of amino acids such as proline and glutamine likely provides organic nitrogen that supports meristematic cell proliferation and regenerative capacity [15,23]. The comparison of WPBS-based media with other formulations derived from *Eragrostis tef* (Media A–C) and MSD (Media D–G) indicates a better performance of the WPBS base under the conditions evaluated. This is consistent with a potential role for mineral and nitrogen balance in supporting regenerative capacity and with the broader recognition that basal medium composition is a key determinant of whether somatic embryogenesis or de novo organogenesis predominates in vitro [24]. The contrasting responses observed between Medium I and Medium J are consistent with patterns reported in warm-season grasses, in which 2,4-D is commonly associated with somatic embryogenesis, whereas BAP is more closely associated with shoot regeneration [12].

Although additional physiological evidence would be required, the present data are consistent with an interpretation in which the basal formulation may support morphogenic competence, while the exogenous growth regulator combination may be associated with the predominant morphogenic response. This conceptual model is consistent with previous studies showing that exogenous plant growth regulator composition can influence regenerative development toward somatic embryogenesis or shoot organogenesis [20,24] and may guide the rational design of regeneration protocols for other recalcitrant grasses.

### 3.3. Exogenous 2,4-D and BAP Concentrations Are Associated with Alternative Morphogenic Responses

The observation of two morphogenically distinct responses—SELSs and multiple-shoot proliferation—in the same apical region of the embryonic axis is a notable finding. SELSs were characterized by their compact white to translucent globular morphology, absence of chlorophyll pigmentation, and lack of progression toward embryo maturation. In contrast, multiple-shoot proliferation resulted in the formation of green, organized shoot clusters that subsequently developed into complete plantlets. These contrasting patterns are consistent with the distinction between somatic embryogenesis, in which somatic embryos develop into whole plants, and organogenesis, in which shoots and roots develop sequentially under appropriate culture conditions [12]. For this reason, the arrested globular structures observed in Group 1 are conservatively referred to as SELSs.

Although the present study evaluated exogenously supplied 2,4-D and BAP, morphogenic responses reflect the interplay between exogenous plant growth regulators and endogenous hormonal regulation, including the spatial organization of auxin within the explant [22]. Therefore, the associations observed here should not be interpreted as indicating that exogenous plant growth regulator concentrations alone determine morphogenic outcomes.

The lack of further development of SELSs may be consistent with reports from other species in which reduction or removal of 2,4-D was required for subsequent somatic embryo development [15,23], although the developmental identity of SELSs remains to be histologically established.

Comparable alternative morphogenic responses have also been described in other plant species. Particularly relevant is the report in the grass *Pogonatherum paniceum*, in which mature caryopsis-derived cultures exhibited somatic embryogenesis, shoot organogenesis, or both developmental capacities depending on the concentration of 2,4-D [20]. By contrast, in sorghum, these two developmental pathways were reported to seldom occur simultaneously within the same explant [21], suggesting that the frequency of coexistence between morphogenic responses may vary among grass species and genotypes.

### 3.4. Light Intensity Modulates Morphogenic Responses

A relevant finding of this study is the effect of light intensity during the induction phase. The systematic evaluation of four light intensities revealed that light intensity significantly affected both morphogenic induction and multiple-shoot proliferation, with the light intensity associated with the highest response differing between the two morphogenic responses. For morphogenic induction in Group 1, medium light (55.2 µmol m^−2^·s^−1^) produced the highest response in most media, although for Medium I, the best-performing formulation, responses were comparable across all three illuminated intensities. In contrast, multiple-shoot proliferation was promoted by medium and high light intensities, with Medium J achieving 74.0% under medium light and Medium K reaching 59.4% under high light.

The consistently lower responses observed under darkness across most culture media contrast with the conventional use of dark incubation for grass callus induction [3]. These findings suggest that light during the induction phase may contribute to maintaining morphogenic competence and supporting regeneration in mature *E. curvula* embryos.

Moreover, the distinct light responses observed for SELS-associated morphogenic induction and multiple-shoot proliferation suggest that these two morphogenic responses may differ in their photomorphogenetic requirements, consistent with previous evidence that light can modulate plant regeneration and morphogenic responses to exogenous growth regulators [16,18,25].

Plant regeneration is a complex process influenced by multiple interacting factors. Within the experimental conditions evaluated here, the significant medium × light interaction detected by the GLM indicates that the effect of light intensity on morphogenic response varied with the induction-medium formulation. This interaction underscores the importance of considering both nutritional and environmental factors simultaneously when optimizing regeneration protocols.

### 3.5. Implications for Regeneration and Genetic Transformation

The protocols developed in this study provide a reproducible regeneration framework that may support future *Agrobacterium*-mediated or biolistic transformation of *E. curvula*. The identification of two candidate target tissues—mature embryo shoot apices at 2–3 days of culture and multiple shoot clusters after 3–4 weeks— provides two potential entry points for future transformation studies. The ability to obtain morphogenic responses of up to 60.6% in Group 1 and multiple-shoot proliferation of up to 74.0% in Group 2 from mature embryos provides a solid basis for future transformation studies. Regenerated plantlets developed well-formed shoots and root systems in vitro, indicating suitability for transfer to *ex vitro* conditions. Efficient regeneration systems constitute the foundation for downstream applications, including micropropagation, genetic transformation, and genome editing in warm-season grasses [12].

Together, these results provide a reproducible regeneration framework and identify the mature embryo shoot apex as a suitable target tissue for future genetic transformation and genome editing strategies in *E. curvula*.

### 3.6. Limitations and Future Perspectives

Several limitations of this study should be acknowledged. First, the developmental identity of the globular structures was inferred from external morphology and was not confirmed histologically. Although the morphological characteristics observed are consistent with descriptions reported in previous studies of grasses [1,14], histological analyses will be required to establish their anatomical organization and developmental identity, including whether SELSs exhibit bipolar organization and an independent vascular system lacking vascular continuity with the maternal tissue [23]. Until such analyses are performed, their embryogenic identity cannot be confirmed.

Second, SELSs and associated shoot-forming meristematic tissue were not quantified as separate response categories within Group 1 media. Consequently, the statistical analyses evaluated overall morphogenic responsiveness rather than the independent contribution of each morphogenic component. Future studies should quantify these responses independently to determine how each responds to culture medium composition and light intensity and to better resolve their relative contributions to the overall morphogenic response.

Third, although overall morphogenic responses were quantified, the regeneration capacity of individual SELSs was not assessed. Future studies should determine the efficiency with which SELSs can develop into complete plants.

Fourth, the molecular basis of the differential morphogenic responses observed across light intensities and culture media remains to be elucidated. Transcriptomic and proteomic analyses of embryonic shoot apex tissues under different media and light conditions could provide insights into the regulatory networks underlying regenerative capacity and morphogenic responses.

Finally, the study was conducted with a single genotype, Tanganyika INTA. The applicability of these protocols to other *E. curvula* genotypes remains to be determined. Future work should evaluate the protocols developed here across a broader range of genotypes.

## 4. Materials and Methods

### 4.1. Plant Material and Surface Sterilization

Mature seeds of *E. curvula* cv. Tanganyika INTA were used as the starting material. Seeds were placed in 1.5 mL Eppendorf tubes and surface-sterilized as follows. They were washed three times with distilled water containing a few drops of Tween 20 (PhytoTechnology Laboratories, LLC, Lenexa, KS, USA), treated with 2.5% (*v*/*v*) hydrogen peroxide (H_2_O_2_) for 5 min, and incubated in 70% (*v*/*v*) ethanol for an additional 10 min. Seeds were then exposed to 7% (*v*/*v*) sodium hypochlorite solution for 15 min. Finally, under a laminar flow hood, the seeds were rinsed five times with sterile distilled water.

### 4.2. Explant Preparation

Mature embryos were extracted from the surface-sterilized seeds using hypodermic needles; this approach allowed for precise dissection from the endosperm. The isolated embryos were placed on the culture medium in Petri dishes with the scutellum facing upward, and were used as the sole explant type, with the apical region of the embryonic axis, including the shoot apical meristem (SAM), as the target tissue.

### 4.3. Induction Media

Eleven media (A–K) were designed for cv. Tanganyika INTA and organized into two functional groups (Table 1). The first group (A–I) was designed for embryogenic callus induction, whereas the second group (J and K) was designed for multiple-shoot proliferation.

Media A–C were based on the optimized callus induction medium described by Gugsa and Kumlehn [14] for immature embryos of *Eragrostis tef*, which contained KBP mineral salts, 6 mM glutamine, 0.5% Phytagel™, MES buffer, and the complex organic supplement described by Kao and Michayluk [26]. Several components of the original formulation were retained: glutamine (877 mg/L, 6 mM), Phytagel™ (0.5%), MES (1.9 g/L), and maltose (3% *w*/*v*) as the carbohydrate source. However, several substantial modifications were introduced: KBP minerals were replaced with MS salts [27], and Gamborg B5 vitamins [28] were used instead of the complete Kao and Michayluk supplement, while four components of the latter were retained: biotin (0.01 mg/L), riboflavin (0.08 mg/L), calcium pantothenate (1.0 mg/L), and ascorbic acid (0.8 mg/L). These components were retained from the reference formulation, and the concentrations reported here correspond to the amounts added to the medium. Ascorbic acid is known to decay rapidly in plant tissue culture media [29], and its persistence in the medium during culture was not determined in this study. Riboflavin and ascorbic acid were present at lower concentrations than in the original formulation. This formulation contained 2 mg/L 2,4-D and no BAP and was designated Medium A. Medium B was derived from Medium A by increasing the 2,4-D concentration to 5 mg/L, while Medium C was formulated by adding 500 mg/L casein hydrolyzate to Medium A.

**Table 1:** Induction media used in this study for *Eragrostis curvula*. Media A–C were based on Gugsa and Kumlehn [14], Media D–G on the MS-based MSD formulation [30], Media H–J on WPBS-B [10], and Medium K on the SM 2.5 shoot multiplication medium [9].

Medium	Base Formulation	2,4-D (mg/L)	BAP (mg/L)	Modifications Relative to Reference Formulation
**A**	Based on Gugsa and Kumlehn [14]	2	0	KBP salts replaced with MS salts; Kao and Michayluk supplement replaced with B5 vitamins, with biotin and calcium pantothenate retained and riboflavin and ascorbic acid provided at reduced concentrations.
**B**		5	0	As Medium A, with 2,4-D increased from 2 to 5 mg/L
**C**		2	0	As Medium A, with 500 mg/L casein hydrolyzate added
**D**	Based on MSD (MS-based) [30]	2	0.01	MS vitamins replaced with Gamborg B5 vitamins; 0.01 mg/L BAP added
**E**		2	0	As Medium D, but without BAP
**F**		2	0.01	As Medium D, with maltose increased from 3% to 6%
**G**		2	0	As Medium D, with maltose increased from 3% to 6% and without BAP
**H**	Based on WPBS-B [10]	2	0	-
**I**		2	0.01	As Medium H, with 0.01 mg/L BAP added
**J**		0.5	2	As Medium H, with 2,4-D reduced from 2 to 0.5 mg/L and BAP increased from 0 to 2 mg/L
**K**	SM 2.5 (MS basal medium + casein hydrolyzate) [9]	0.5	2	-

Media D–G were derived from the MSD medium described by Souza Canada et al. [30], based on the formulation of Barcelo and Lazzeri [31], which contains maltose as the carbohydrate source. Two modifications were introduced in our adaptation: (i) replacement of MS vitamins with Gamborg B5 vitamins [28], and (ii) addition of a low concentration of BAP (0.01 mg/L) together with 2,4-D (2 mg/L). Because the B5 vitamin complex lacks glycine, this compound was supplemented separately at 2 mg/L to maintain the same final glycine concentration as in the original MSD formulation. This formulation was designated Medium D. Medium E was derived from Medium D by omitting BAP. Medium F was formulated by increasing maltose concentration to 6% (*w*/*v*) while retaining BAP, and Medium G was formulated by increasing maltose to 6% (*w*/*v*) without BAP.

Media H, I, and J were based on the WPBS-B formulation described by Dalton [10], a modification of MS medium with optimized mineral and nitrogen composition for improved embryogenesis and morphogenesis. Medium H corresponded to the WPBS-B callus-induction formulation described by Dalton [10], containing 2 mg/L 2,4-D and no BAP. Medium I was derived from Medium H by adding a low concentration of BAP (0.01 mg/L), whereas Medium J was formulated by reducing 2,4-D to 0.5 mg/L and increasing BAP to 2 mg/L. Medium K was based on the shoot multiplication medium (SM2.5) described by Ahmad et al. [9] for wheat, consisting of MS basal medium supplemented with 500 mg/L casein hydrolyzate, 2 mg/L BAP, 0.5 mg/L 2,4-D, and 3% sucrose.

All media were adjusted to pH 5.8 prior to autoclaving, except for Media H–J, which were adjusted to pH 5.6. Heat-labile components, including amino acids, vitamins and plant growth regulators, were filter-sterilized (0.22 μm) and added to the medium after autoclaving.

Unless otherwise stated, all chemicals and plant growth regulators used for culture-medium preparation were purchased from Sigma-Aldrich/Merck (Darmstadt, Germany).

### 4.4. Culture Conditions and Light Treatments

Cultures were maintained in a growth chamber at 25 ± 2 °C. Illumination was provided by 30 W fluorescent horticultural tubes (Gro-Lamp, Interlec, Buenos Aires, Argentina) mounted above the culture shelf at a fixed distance of 24 cm. Four light regimes were evaluated by varying the number of fluorescent tubes: one tube for low light, two tubes for medium light, and four tubes for high light, all under a 16/8 h (light/dark) photoperiod, whereas the fourth regime was complete darkness. Illuminance was measured at shelf level using a digital lux meter (TES-1330A, TES Electrical Electronic Corp., Taipei, Taiwan) as the mean of three independent readings for each lighting configuration, and converted to photosynthetic photon flux density (PPFD) using a lamp-specific lux-to-PPFD conversion factor of 0.030 µmol m^−2^·s^−1^ per lux. The resulting illuminances of 980, 1840, and 3580 lux corresponded to PPFD values of 29.4, 55.2, and 107.4 µmol m^−2^·s^−1^ for the low-, medium-, and high-light treatments, respectively.

For each treatment, 24–37 mature embryos were cultured in each Petri dish. The number of biological replicates ranged from three to six, depending on explant availability across independent experiments. Each Petri dish was considered an independent experimental unit. Responsive (YES) and non-responsive (NO) explants were recorded separately for each dish, and dish-level binomial counts were used for statistical analysis. Explants were maintained on induction medium for approximately 8 weeks. Cultures were regularly inspected for signs of visible microbial contamination. Localized contamination restricted to the culture medium was carefully removed under aseptic conditions when possible; visibly contaminated explants were removed, and cultures with extensive contamination were discarded. Only evaluable explants were included in the final counts of responsive (YES) and non-responsive (NO) explants used for statistical analysis.

### 4.5. Morphological Assessment and Structure Identification

SELSs were identified descriptively based on their compact, globular morphology and white-to-translucent appearance. Their compact, globular morphology is consistent with features reported for embryogenic cultures of graminaceous species [32]. Histological analysis was not performed; therefore, the term SELS is used exclusively as a morphological descriptor and does not imply histologically confirmed somatic embryogenesis. Bipolar organization and the presence or absence of vascular connection to the maternal tissue, which are features relevant to distinguishing somatic embryogenesis from organogenesis [23], were not determined. Multiple shoots were identified as clusters of green, organized shoot-like structures emerging from the apical region of the embryonic axis, with visible leaf primordia and a defined apical dome [8,9]. All observations were performed using a stereomicroscope (SZM-LED2, OPTIKA Microscopes, Ponteranica, Italy) at magnifications ranging from 7× to 45×. The development of structures was monitored regularly from the first to the last week of the induction period (as detailed in Figure 1) to track their origin and progression. High-resolution photographs were taken at each stage using a digital camera (MIKOBA 900, model CM900) attached to the trinocular port via the phototube (Figure 1).

### 4.6. Assessment Criteria and Statistical Analysis

Induction efficiency (%) was defined as the proportion of responsive explants among the total number of evaluable explants. In Group 1 (Media A–I), responsiveness was defined as the formation of SELSs and/or associated shoot-forming meristematic tissue, whereas in Group 2 (Media J and K), it was defined as multiple-shoot formation. For each treatment combination (culture medium × light intensity), induction efficiency was calculated as a proportion of responsive explants, expressed as YES/(YES + NO), where YES and NO represent the numbers of responsive and non-responsive explants, respectively. The raw experimental data used for all generalized linear model analyses are provided in Supplementary Table S1.

For each functional group, the numbers of responsive and non-responsive explants per Petri dish were analyzed using generalized linear models (GLMs) with a binomial error distribution and logit link function in R version 4.5.0. Culture medium, light intensity, and their interaction were included as fixed effects in the models. Analyses were performed separately for Group 1 (Media A–I; morphogenic responses characterized by SELS formation and/or associated shoot-forming meristematic tissue) and Group 2 (Media J and K; multiple-shoot proliferation).

Type III analyses of deviance were performed using the Anova function from the car package (version 3.1-3), and statistical significance was assessed using likelihood-ratio chi-square (LR χ^2^) tests. Estimated marginal means (EMMs) were obtained using the emmeans package (version 2.0.3) on the response scale (predicted probabilities), and pairwise comparisons were adjusted using the Sidak method. Compact letter display (CLD) groups were generated using the multcomp package (version 1.4-30) to identify statistically homogeneous treatment groups at α = 0.05. Predicted probabilities and their 95% confidence intervals were used to construct Figure 4. Complete model outputs, estimated marginal means, compact letter displays, and the raw experimental dataset are provided in Supplementary Tables S1–S7.

## 5. Conclusions

The mature embryo shoot apex constitutes a suitable explant for *E. curvula* regeneration. Distinct morphogenic responses were observed under different combinations of culture medium composition and light intensity, resulting in either somatic embryo-like structures or multiple-shoot proliferation. The results are consistent with a conceptual model in which the WPBS formulation may provide a nutritional context that supports morphogenic competence, whereas differences in exogenous 2,4-D and BAP concentrations and light intensity are associated with distinct morphogenic outcomes. This model should be considered a conceptual model that requires further experimental validation. The optimized protocols described here identify the mature embryo shoot apex as a suitable target tissue and provide a reproducible platform for future Agrobacterium-mediated transformation, biolistic transformation, and genome editing of this important forage grass.

## Data Availability

Data presented in this study are available in the article and Supplementary Materials.

## Supplementary Materials

The following supporting information can be downloaded at https://doi.org/10.5281/zenodo.21715769, Table S1: Petri dish-level experimental data used in the generalized linear model analyses. Each row represents one independent Petri dish. Responsive (YES), non-responsive (NO), and total explants were recorded separately for each dish; Table S2: Analysis of deviance (Type III likelihood-ratio chi-square (LR χ^2^) tests) for the generalized linear model fitted to Group 1 (SELS-associated morphogenic induction); Table S3: Analysis of deviance (Type III likelihood-ratio chi-square (LR χ^2^) tests) for the generalized linear model fitted to Group 2 (multiple-shoot proliferation); Table S4: Observed response percentages and estimated marginal means (predicted probabilities, standard errors, and 95% confidence intervals) for Group 1 (SELS-associated morphogenic induction); Table S5: Observed response percentages and estimated marginal means (predicted probabilities, standard errors, and 95% confidence intervals) for Group 2 (multiple-shoot proliferation); Table S6: Compact letter display summarizing Sidak-adjusted pairwise comparisons for Group 1; Table S7: Compact letter display summarizing Sidak-adjusted pairwise comparisons for Group 2; Figure S1: Representative photographs illustrating the morphological changes described in Section 2.1; Figure S2: Representative examples of SELS-associated morphogenic responses observed in Group 1 culture media; Figure S3: Representative examples of multiple-shoot proliferation observed in Group 2 culture media; Original Uncropped Images for Figure 1, Figure 2 and Figure 3; Supporting Images for Figure 1, Figure 2 and Figure 3.
